# Facilitators and barriers to nurses’ compliance with continuous professional development requirements at a referral hospital in Oshana Region, Namibia

**DOI:** 10.4102/curationis.v48i1.2637

**Published:** 2025-02-19

**Authors:** Rauha Hamukoto, Daniel O. Ashipala, Phellep N. Muhora, Julia Amadhila

**Affiliations:** 1 Department of General Nursing Sciences, School of Nursing and Public Health, Faculty of Health Sciences and Veterinary Medicine, University of Namibia, Rundu, Namibia; 2 Department of General Nursing Sciences, School of Nursing and Public Health, Faculty of Health Science and Veterinary Medicine, University of Namibia, Oshakati, Namibia

**Keywords:** facilitators, nurses, barriers, continuous professional development, compliance

## Abstract

**Background:**

Health professionals must keep their knowledge, skills and ethics up to date to ensure competency and promote the public interest, safety and health of all Namibians. In Namibia, little research exists on the facilitators of, and barriers to, nurses’ compliance with continuous professional development (CPD) requirements.

**Objectives:**

The objective of this study was to explore and describe the facilitators of, and barriers to, nurses’ compliance with CPD requirements at Intermediate Hospital Oshakati (IHO) in the Oshana region of Namibia.

**Method:**

Purposive sampling was used in this qualitative, explorative, descriptive and contextual research study to select participants who met the inclusion criteria. Data were collected via individual semi-structured interviews with 15 of the 550 nurses employed at the hospital, at which point data saturation was reached. The transcribed data were then analysed using thematic analysis. The collected data were analysed thematically using an inductive approach.

**Results:**

The findings were synthesised under three themes: facilitators of nurses’ participation in CPD, barriers to nurses’ participation in CPD and recommendations for improvements.

**Conclusion:**

Time constraints, a shortage of nurses, limited access to digital technologies, a lack of funding for CPD training and a lack of accredited CPD service providers are major barriers to participation in CPD training. Strengthening communication and collaboration between health workers, CPD providers and management are specifically organisational factors seen as crucial to successful staff development.

**Contribution:**

The findings from this study can be used to create some targeted interventions and ongoing strategies to enhance nurses’ compliance with CPD requirements.

## Introduction

The role of medical professionals keep changing because of technological advancements and ever-changing demographics health issues, with new ways of handling medical cases constantly being discovered. Continuous professional development (CPD) helps health professionals to maintain, improve and increase their knowledge, expertise and competence when managing medical cases (Macaden et al. 2017). Spear (2016) defined CPD as a process of improving and increasing nursing competencies and knowledge through access to nursing education and training opportunities in the workplace or through the observation of other nurses performing nursing tasks. The CPD assists nurses during their transition into practice to improve their competencies in order to provide expert care to their patients (Karami, Farokhzadian & Foroughameri 2017). According to Daniels and Jooste (2018), competencies reflect professional recognition and the ability of nurses to deliver a specified professional service by effectively using their cognitive, affective and psychomotor skills. The way diseases such as tuberculosis used to be managed 5 years ago has changed, which is why it is critical that nurses stay abreast of new ways of working and providing treatment.

Professional regulatory bodies oversee CPD to make sure that healthcare workers follow set standards. Belgium and the United Kingdom (UK) are two countries that have made CPD mandatory for healthcare professionals (Watson 2021). In the United States, where CPD has matured substantially over the past two decades, CPD is used to ensure that health workers participate in ongoing learning and are aware of the latest clinical developments (Sherman & Chappell 2018). Nigeria is an example of an African country that has introduced mandatory CPD programmes to keep nurses up to date in terms of their education and abilities (Adeyemo & Ajibade 2022).

Sherman and Chappell (2018) found that in some countries, CPD has moved from single profession educational designs and formal didactic methods of delivery to educational models that are innovative, dynamic and learner centric. Ryder et al. (2018) agreed, noting that the provision of first-rate nursing care, such as personal care, homemaking, and companionship, often requires healthcare workers to stay abreast of research-backed education and follow high standards of practice. Unfortunately, CPD in some hospitals and countries is not given the requisite attention (Sherman & Chappell 2018), including at the Intermediate Hospital Oshakati (IHO) in Namibia. In these cases, nurses are obliged to continue learning about current health trends in healthcare such as patient care, treatments and techniques throughout their careers (Day et al. 2018).

As nursing practice is dynamic and rapid changes are happening in healthcare systems, the Health Professions Council of Namibia (HPCNA) committed in 2011 to implementing a CPD system as a means to aspire to excellence in healthcare provision and delivery. According to the HPCNA, the purpose of CPD is to assist healthcare professionals to acquire and maintain new and updated levels of knowledge, skills and ethical attitudes, which will be of measurable benefit in professional practice, as well as to promote and enhance professional integrity. The beneficiary will ultimately be the patient/client. The Namibian Health Professions legislation has prescribed CPD as the means for maintaining and updating professional competence to ensure that the public interest will always be promoted and protected, as well as to guarantee the best possible service to the community.

The HPCNA recommended that, as of 2011, every healthcare professional registered in Namibia be required to accumulate continuing education units (CEUs) (30 for professional groups and 15 for supplementary groups) per 12-month period, of which at least five should cover ethics, human rights and medical law (HPCNA 2011). It further endorsed that a CPD system be put in place for all Councils within the HPCNA, as CPD is both an ethical and a statutory responsibility. Against this backdrop, Palma, Oducado and Palma (2020) stated that continual learning or CPD is not just a nurse’s personal and professional responsibility, but a legal and ethical duty that they owe to themselves and others. As Mlambo, Silén and McGrath (2021) stated, CPD is central to nurses’ lifelong learning and is vital for keeping nurses’ knowledge and skills up to date.

While CPD is mandatory for every registered healthcare professional in Namibia, 82% of Namibian nurses did not submit the required CEUs to the HPCNA (HPCNA 2011). One of the rules of the HPCNA is that those who do not attain the required CPD sessions are considered to be outdated (HPCNA 2011). This study thus sought to investigate the facilitators of, and barriers to, the participation of nurses at IHO in CPD.

As CPD is the key tool for maintaining and updating the professional competencies of nurses, all registered and enrolled nurses are required to comply with CPD requirements. Despite this, by 2011, 55% of registered nurses in Namibia did not attain the required CEUs (HPCNA 2011). This non-compliance seems to be attributed to a number of factors, including a lack of motivation, a lack of support from hospital management and a lack of understanding of the importance of CPD compliance for the professional development of nurses (HPCNA 2011). According to the Namibian Patient Charter, patients expect to be treated by professional nurses who are skilful, knowledgeable and competent and nursing care should take place in a safe environment (Ministry of Health and Social Services 2016). As non-compliance with CPD means that nurses will not improve their knowledge or their intellectual and personal skills and self-confidence, one of the rules of the HPCNA is that only those who attain the required CPD points are eligible for future employment opportunities (HPCNA 2011). A study conducted by Newaka, Pretorius and Josua (2022) reported that staff shortages, a lack of hospital support, professional growth and networking were some of the factors that influence registered nurses’ compliance with CPD requirements.

Although the coordination and facilitation of CPD activities at the IHO has been ongoing since 2010, it would appear that the nurses do not have a positive view of their participation in CPD activities. Nurses who are not given access to CPD might find themselves dissatisfied with their work, which results in a lower standard of care and making it more difficult to recruit and retain nursing staff (Watson 2021). This research thus investigated the facilitators of, and barriers to, the participation of nurses in terms of the CPD requirements at IHO and compared these with other hospitals around the world. This research is critical, as offering ongoing CPD in healthcare is vital to make sure that all nurses are able to provide safe, reliable services (Manley et al. 2018). Many studies have been conducted on CPD, some of which have focused on the awareness and attitudes of nurses to CPD (Mnguni 2018; Palma et al. 2020). Others have focused on CPD needs, as well as facilitators of and barriers to CPD for registered nurses only (Macaden et al. 2017).

### Problem statement

Despite the significance of CPD initiatives in enhancing the skills and knowledge of nurses, 55% of registered nurses at IHO did not attain the required CEUs (HPCNA, 2011). This non-compliance to CPD requirements could imply that there are barriers that are being overlooked in the implementation of CPD programmes that need urgent attention, as a lack of compliance might lead to a decline in the quality of patient care. No studies have focused on the facilitators of, and barriers to nurses’ compliance with CPD requirements in Namibia. One CPD study conducted focused on the effort and reward imbalance factors motivating Namibian professional nurses to participate in CPD (Mbidi & Damons 2020). A study by Mbidi & Damons reported four effort-reward imbalance factors, namely: (1) extrinsic efforts, (2) intrinsic efforts, (3) reward motives and (4) over-commitment motives. This dearth of literature led to the need for supplementary research in the Namibian context, which is key to highlighting the factors that will improve compliance with CPD among nurses. This study, which extends to managerial influence on nurses’ compliance with CPD, will not only enhance compliance but also improve the understanding of coordination and management of CPD providers and nurses.

### The objectives of the study were to

- explore the facilitators of, and barriers to, nurses’ compliance with CPD requirements at IHO in Oshana region, Namibia
- describe the facilitators of, and barriers to, nurses’ compliance with CPD requirements at IHO in Oshana region, Namibia.

### Aim of the study

The aim of the study was to explore the facilitators of, and barriers to, nurses’ compliance with CPD requirements at IHO in Oshana region, Namibia.

## Methods

### Study design

This qualitative, exploratory, descriptive and contextual research study was guided by a semi-structured interview guide, which was developed to explore and describe the facilitators of, and barriers to, nurses’ compliance with CPD requirements at IHO in Oshana region, Namibia. A qualitative research method was utilised because it allowed the researcher to explore a topic with limited extant research. Such a design explores how people make sense of their surroundings, experiences and understandings of phenomena (Green & Thurgood 2018). The purpose of a descriptive design is to document and describe the phenomena of interest (Marshall & Rossman 2016), which, in this study, were the facilitators of, and barriers to, nurses’ compliance with the CPD requirements at IHO. In addition, the method used in the study illuminated how these phenomena have come about and helped to uncover the full nature of this little-understood concern (Polit & Beck 2017). The facilitators of, and barriers to, nurses’ CPD compliance were described in relation to IHO, hence this study was contextual in nature. Consolidated criteria for reporting qualitative research (COREQ) were used when preparing the manuscript (O’Brien et al. 2014).

### Study setting

This study was conducted at IHO in Oshana region, Namibia. The hospital is situated in the north of Namibia, approximately 550 km from Windhoek. It serves a total population of 37 000 people and has a 750-bed capacity and a staff complement of 550 nurses (enrolled and registered). Intermediate Hospital Oshakati serve as a referral hospital for five district hospitals in the three north west regions: Ohangwena, Omusati and Oshikoto. It is important for healthcare professionals working at IHO to be well versed in terms of their knowledge, skills and ethics in order to ensure competency and promote the public interest, safety and health of all Namibians.

### Study population and sample

The targeted population for this study was registered and enrolled nurses. Purposive sampling was used to recruit nurses for the study, utilising the following inclusion criteria: a registered or enrolled nurse should be working at the IHO for at least 1 year, being available at the time of the interviews and being willing to participate by signing an informed consent form. Nursing students or nurses from other hospitals were excluded from the study.

### Data collection

Semi-structured individual interviews were conducted during December 2020 at the IHO. Before the main data collection, the authors conducted a pilot study with three nurses to establish whether there were any problems with the data collection tool that would need to be addressed (Polit & Beck 2018). The data collected during the pilot study were not incorporated into the main study. After dates were agreed upon for the interviews, the participants were able to choose a venue and time that suited them. The interviews were held in a private room to ensure confidentiality and quiet, as well as to not disrupt the hospital’s daily routines and activities.

An information sheet was distributed by hand to the participants before their interviews to inform them of the reason for the study, their rights as participants, and how the study would take place. Having received written consent from the respondents, the researcher used an interview guide to conduct the in-depth individual interviews until data saturation was reached, at the 15th participant. All of the interviews were undertaken in English, which is one of the official languages of Namibia. Each of the participants was comfortable using this language. The time for each interview was estimated to be between 45 and 59 min to allow participants enough time to express their views regarding the phenomena under study. When necessary, field notes were taken during the interviews, which were followed up by probing, open-ended questions to ensure the clarity of the participants’ responses. The field notes were combined with the transcribed interviews to ensure detailed information. With the permission of the participants, the interviews were tape recorded to enable the researcher to refer back to them during transcription of the data.

The interview guide consisted of the following questions, which were asked during the interviews:

- What are the facilitators of nurses’ compliance with CPD requirements at IHO?
- What are the barriers to nurses’ compliance with CPD requirements at IHO?
- What should be done to help nurses in the IHO to be compliant with CPD training?

### Data analysis

Following the interviews, the researcher listened to the audio recordings of each interview repeatedly before transcribing the audio content into text (Braun et al. 2019). All the data collected were then analysed to give them meaning (Grove, Burns & Gray 2019). Constant comparison analysis was used to extract the emerging themes and sub-themes from the data, with the four coding phases (initial, focused, axial and theoretical) being used. In the initial coding phase, line-by-line coding was performed manually using the process coding method by Saldana (2009). This was followed by a cross-transcription comparison, which was conducted in the focused coding phase to identify the most frequent and significant codes. The codes were linked to subcategories, properties and dimensions in the axial coding phase, and the main categories for the themes were identified in the final theoretical coding phase (Braun et al. 2019).

### Study quality criteria

Credibility, dependability, transferability and confirmability were ensured to achieve trustworthiness (Speziale, Streubert & Carpenter 2011). Credibility was achieved through prolonged engagement, with one researcher spending a month in the clinical setting in which the research was conducted, and both individual interviews and field notes were used as methods of data collection. In addition, member checking was carried out, with the researchers constantly checking their findings with the participants and comparing them with the literature review. Transferability was achieved through dense description, which included a comprehensive description of the research methods used and the direct quotes of participants for illustration purposes. Dependability was achieved through an audit trail, which is available upon request. In order to confirm the data, a consensus meeting with an independent coder, who was experienced in qualitative research and held a doctorate in nursing education, was utilised. Additionally, verbatim transcriptions of the interviews and re-reading of these and the field notes enabled the researcher to get a better understanding of the phenomena under investigation.

### Ethical considerations

The research was conducted after approval was granted by the School of Nursing and Public Health Research and Ethics Committee of the Faculty of Health Sciences and Veterinary Medicine. The researcher obtained permission to conduct the study from the University of Namibia (ethical clearance number: SoNEC 02/2021), as well as from Ministry of Health and Social Services (Ref: RPH/2020) before the study was conducted. The research was undertaken according to the Declaration of Helsinki. The first author communicated with the hospital nurse manager telephonically to inform them about the proposed study and what would be required of the participants. The manager then provided the author with the contact details of the unit managers, who in turn supplied the contact details for all the nurses in their respective departments. The participants provided informed consent by signing a form prior to their participation in the study. In addition, the interviewees were aware that their participation was voluntary and that they could withdraw at any time without any penalties. The inclusion and exclusion criteria described above were adhered to, to ensure fairness and avoid bias. No names were required or asked during the interview, with numbers being allocated to each participant instead. Furthermore, to maintain confidentiality, all the audio recordings were stored on a computer file that was locked with a password known only to the researchers.

## Results

### Participant demographics

Fifteen nurses from IHO participated voluntarily in the individual interviews. The high number of females compared to males can be attributed to the fact that the nursing profession in Namibia has more females than males. The demographic characteristics of the 15 participants are shown in Table 1.

**TABLE 1:** Demographic characteristics of the participants (*N* = 15).

Variable	*n*
**Age (years)**	
18–29	3
30–39	5
40–49	4
50 and above	3
**Gender**	
Male	6
Female	9
**Marital status**	
Single	4
Married	11
**Working experience (years)**	
1–10	5
10–20	6
20–30	4

### Presentation of findings

The three themes that emerged from the data collection were: personal understanding of CPD, facilitators of nurses’ participation in CPD, barriers to nurses’ participation in CPD and recommendations for improvements. Table 2 summarises the study findings in the form of the themes and sub-themes that emerged from the data analysis.

**TABLE 2:** Themes and sub-themes.

Themes	Sub-themes
Facilitators of nurses’ participation in CPD	Knowledge of current health trends
	Conformity to professional regulatory requirements
	Skills competency
	Access to technology
	Managerial and peer enthusiasm
Barriers to nurses’ participation in CPD	Time constraints
	Shortage of nurses
	Limited access to tools/technology
	A lack of funding for CPD training
	A lack of nurses with expertise to train and lack of accredited CPD service providers
Recommendations for improvements	Develop a database for in-service training
	Provide on-line CPD training
	Make funds available for CPD training

CPD, Continuous professional development.

### Theme 1: Facilitators of nurses’ participation in continuous professional development

This theme reflects certain factors that motivate nurses to participate in CPD, including knowledge of current health trends, conformity with professional regulatory requirements, skills competency, access to technology, as well as manager and peer enthusiasm.

#### Knowledge of current health trends

The participants indicated that what motivates them to participate in CPD is keeping up to date with the ‘need to know’ health trends so they know how to treat patients appropriately. For instance, in the coronavirus disease 2019 (COVID-19) era, without participating in CPD, nurses would not know when to suspect it and how to act after suspecting a case:

‘We attend because we want to know new things about diseases, medications and how to attend to specific conditions.’ (P5, 32 years, female)

‘New diseases are coming especially these days and you can only know much about it through in-service trainings.’ (P4, 28 years, female)

#### Conformity to professional regulatory requirements

The participants revealed that what mostly drives them to participate in CPD is that they are required to submit some evidence to the Nursing Council that they have attended in-service training; otherwise they will be suspended from work:

‘You have to attend the training because of the points that are required by the nursing council and if do not submit your CPD points your name will be removed from the roll.’ (P1, 24 years, female)

‘My name was picked at the Nursing Council of Namibia to see if I have complied with CPD requirements which I did not meet by then and I was cautioned to try and meet the required CEUs otherwise my name will removed from the roll. From there I tried by all means to conform to the set CPD requirements.’ (P2, 26 years, male)

#### Skills competency

The participants indicated that in-service training is carried out mainly to improve the competencies of nurses, so they gain more skills. They stressed that nurses cannot rely on the skills they gained during their initial training, as the world is evolving and technological advancements are being introduced every day:

‘New machines are being introduced and nurses need to be trained on how to use them.’ (P2, 26 years, male)

‘In-service training is crucial because nurses are being taught new approaches to nursing care delivery as things keep changing.’ (P6, 36 years, male)

#### Access to technology

One participant highlighted that most nurses find it easy to participate in CPD because most training is performed online; therefore they can attend in the comfort of their homes:

‘It’s easy to attend even when I am off; I just log in with my phone.’ (P2, 26 years, male)

#### Managerial and peer enthusiasm

The participants indicated that most nurses participate in CPD because their role as nurse managers requires them to attend training, while others go for fun or as a way of relieving themselves of the pressures of the ward:

‘When you are a nurse manager you have to attend in-service training.’ (P1, 24 years, female)

‘Other colleagues go there because they want to run away from pressure in the ward, especially when the ward is full.’ (P3, 30 years, male)

### Theme 2: Barriers to nurses’ participation in continuous professional development

This theme is a description of what has been observed by nurses as hindrances to full participation in CPD. These include time constraints, a shortage of nurses, limited access to tools/technologies, a lack of funding for training, a lack of nurses with expertise to train other nurses and a lack of accredited CPD service providers.

#### Time constraints

According to the participants, the major barrier to participation in in-service training is a lack of time, for example, most training is performed in the morning when nurses are busy with their morning routines in the wards:

‘We cannot leave patients unattended going for training.’ (P1, 24 years, female)

‘We will be busy with giving medication, doctors’ rounds and washing patients in the morning so we cannot attend the in-service training if they do it in the morning.’ (P4, 28 years, female)

‘We usually do not get time because they do the training in the morning when the ward is busy.’ (P10, 45 years, female)

#### Shortage of nurses

The participants revealed that a shortage of staff is a major factor that prevents them from attending in-service training, that is, they cannot leave the ward because there will be no one to take care of the patients:

‘We cannot go for training; no one will attend to patients.’ (P11, 48 years, male)

‘The patients are a lot and nurses are few so everyone should stay in the ward.’ (P12, 47 years, female)

‘How then can we go for in-service training when we are not even enough to give patients the care they require?’ (P14, 58 years, female)

#### Limited access to tools/technology

Some participants highlighted that they fail to attend in-service training because it is generally announced on social media platforms, and they do not have the necessary access to these platforms. As a result, they are unable to attend in-service training:

‘They announce the training on WhatsApp and some of us do not have it.’ (P7, 35 years, female)

‘If it is done online I don’t attend because I don’t have a smartphone or computer.’ (P9, 43 years, male)

#### A lack of funding for continuous professional development training

One participant indicated that more funds are needed for in-service training and that money is not always available:

‘Sometimes there are no funds for in-service training.’ (P8, 39 years, female)

#### A lack of nurses with expertise to train and a lack of accredited continuous professional development service providers

The participants explained that too few nurses are accredited to hold CPD training, which is the greatest barrier to participation. They went on to say that it would be better not to attend training at all than to be trained by someone without expertise:

‘Sometimes there is no one to host the in-service training.’ (P6, 36 years, male)

‘There is a lack of accredited CPD service providers, there is no need to attended training of someone who is not accredited.’ (P12, 47 years, female)

### Theme 3: Recommendations for improvements

This theme emerged from the participants’ responses when they were asked to provide their opinions on what can be done to address the barriers to CPD participation. The sub-themes include: develop a database for training, provide online training and make funds available for training.

#### Develop a database for in-service training

The participants suggested that lists of nurses should be drawn up indicating the dates that they would be attending training, as well as the subject, so that every nurse gets an equal chance to participate:

‘They must come up with an in-service training roster of all nurses in all departments to make sure that everyone gets the chance to attend trainings.’ (P7, 35 years, female)

‘Everyone should be given chance to attend in-service training not to only send managers for training yet they are not the one in close contact with the patients, sometimes their feedback is not clear.’ (P13, 51 years, male)

#### Provide on-line continuous professional development training

Some participants suggested that online training should be offered so that everyone can attend a meeting after work or when they are off, as most of them are not able to attend in-service training because of time constraints and the shortage of nurses:

‘They should just encourage everyone to have access to online training.’ (P4, 28 years, female)

‘Online is better; we can even attend when we are off.’ (P11, 48 years, male)

#### Make funds available for continuous professional development training

The participants suggested that the government should provide funding, especially for in-service training:

‘The government should provide money for in-service training.’ (P1, 24, females)

‘More funds should be given to the hospital specifically for CPD training.’ (P8, 39 years, female)

## Discussion

This study was conducted to explore and describe the facilitators of, and barriers to, nurses’ compliance with CPD requirements at IHO in Oshana region, Namibia. The first theme that emerged was ‘Facilitators of nurses’ participation in CPD’, which was related to the nurses’ awareness of current health trends, the need to conform to professional regulatory requirements, skills competency, access to technology and managerial and peer enthusiasm. Certain factors motivate nurses to participate in CPD, including their need to be aware of current health trends so they can act accordingly. For instance, in this COVID-19 era, without participating in CPD, nurses would not know the latest findings regarding the disease. This requires practitioners to update their knowledge and skills regularly to match the changing complexity of healthcare needs (Manley et al. 2018). These findings are similar to those of a study conducted by Viljoen, Coetzee and Heyns (2017), which showed that CPD is necessary for nurses to maintain and build on current knowledge and skills in the rapidly changing healthcare environment.

Continuous professional development plays a crucial role in the ongoing education of nurses and is essential for maintaining their knowledge and skills throughout their careers (Mlambo et al. 2021). The nurses also noticed that they are largely driven to participate in CPD because they are required to submit evidence to the Nursing Council that they are attending in-service training or they may be suspended from work. Similarly, a study conducted by the International Council of Nurses (2021) stated that the CPD law requires all regulated professions, including nursing, to obtain CEUs as a mandatory requirement for the renewal of a professional identification card. This is as per the Health Professions Council of South Africa (HPCSA), which set up the guidelines that nurses must follow, including that it is mandatory that nurses enroll in CPD programmes (HPCSA 2022).

The majority of the participants indicated that in-service training is carried out mainly to improve the competency of nurses and to gain more skills. They further stressed that nurses cannot rely on the skills they gained during their initial training alone, as the world is evolving and technological advancements are being introduced every day. This finding is in line with that of Debska et al. (2020), who stated that CPD ensures that nurses’ professional practice is up to date and contributes to improving patient outcomes and the quality of care.

The study revealed the major barriers to participation in in-service CPD training are time constraints, a shortage of nurses, limited access to tools/technologies, a lack of funding for training, a lack of nurses with expertise to train other nurses and a lack of accredited CPD service providers. As most trainings are carried out in the morning when nurses are busy with their morning routines in the wards, nurses are often unable to attend. A study conducted by Yu, Huang and Liu (2022) similarly found that limited time is an obstacle to CPD. Firstly, authors stated that nursing staff attend CPD activities in their personal time to meet the requirements, which impedes on their family life and vacations. Secondly, participants revealed that a shortage of staff is a major factor that prevents them from attending in-service training, that is, they cannot leave their wards as there are too few nurses and too many patients.

The results of a study conducted by Coventry, Maslin-Prothero and Smith (2015) also revealed that nurses are reluctant to leave or are prevented from leaving the clinical setting to attend CPD owing to a lack of relief cover. This shortage of staff also leads to increasing workloads among nurses, which negatively affects their CPD. Sheikhi et al. (2016) stated that an increasing workload and nursing shortages are some of the factors that affect the ability of nurses to enrol in programmes that will facilitate their professional growth. Other participants highlighted that they fail to attend in-service training because it is usually announced on social media platforms that they do not have access to. Similar results were attained in a study conducted by Swihart and Johnstone (2010), who noted that most professionals miss in-service training because of a shortage of staff in their departments, as well as ineffective ways of communicating about upcoming training.

This study also found that more funds are needed for in-service training. The HPCNA (2011) admitted that CPD training is occasionally interrupted because of a funding shortfall. Finally, it was stressed that there are insufficient numbers of nurses who have the expertise to train others, as well as insufficient accredited CPD service providers, which affects nurses’ ability to attend CPD programmes. According to Mlambo et al. (2021), a lack of CPD-trained nurses, along with inadequate staffing, are the key barriers to formal workplace learning. Similarly, Newaka et al. (2022) reported that the factors that influence nurses’ participation in CPD include financial constraints, a lack of employer support and a shortage of staff.

### Strengths and limitations of the study

The study’s findings were limited by the number of participants interviewed, thus restricting the generalisability of the results. Nevertheless, as this was a descriptive study that provides initial exploratory data, it was not the authors’ intention to generalise. In addition, the study was based on the experiences of nurses working at IHO, thus the study’s findings cannot be generalised to nurses working at other state hospitals.

### Recommendations

This study recognises that there is a need to work with CPD service providers to address these barriers. In addition, the study offers suggestions that could be used by the healthcare sector to ensure more effective CPD training. The study’s findings can further be used to develop managerial strategies and ongoing interventions that focus specifically on addressing the barriers that hinder nurses’ compliance with CPD. Understanding the barriers to, and facilitators of, nurses’ compliance with CPD in the Namibian healthcare system provides valuable insights into the local healthcare context of Oshana region. Identifying these barriers and facilitators has the potential to contribute to the development of targeted interventions and ongoing strategies that are tailor-made to address the barriers faced by nurses at IHO, Oshana Region. Therefore, it is recommended that healthcare institutions consider developing a system to reward nurses who have demonstrated compliance with CPD requirements through the acquisition of 30 CEUs per 12-month period. These rewards could be financial or non-financial, such as promotions, bonuses and/or experience-related payments.

## Conclusion

The study’s aim was to explore the facilitators of, and barriers to, nurses’ compliance with CPD requirements at IHO in Oshana region, Namibia. Despite the facilitators that exist, several barriers must be overcome before CPD training can be fully effective. Time constraints, a shortage of nurses, limited access to tools or technologies, a lack of funding for training, a lack of nurses with the expertise to be trainers and a lack of accredited CPD service providers were recognised by most of the participants as being barriers to their participation in CPD training. The study findings can be used to develop managerial strategies and ongoing interventions that focus specifically on addressing barriers that are hindering nurses’ compliance with CPD.
